# Supplementary material for: Distinct Emotional and Cardiac Responses to Audio Erotica between Genders
Source: Behav Sci (Basel). 2023 Mar 20;13(3):273. doi: 10.3390/bs13030273 (PMC10044842; doi:10.3390/bs13030273)
Supplement: Supplementary file 1 [file behavsci-13-00273-s001.zip › behavsci-2257734-supplementary.pdf]

Table S1: Stimuli (bold) used in this study.

ID	Type	Chi-square		P-value	
		Arousal	Valence	Arousal	Valence
**1**	E	4.29	7.64	**0.75**	0.37
**4**	E	6.16	4.7	**0.63**	0.58
**5**	E	6.63	6.7	**0.47**	0.46
**6**	E	7.97	4.03	**0.44**	0.67
**8**	E	8.17	2.96	**0.41**	0.81
**10**	E	6.13	7.11	**0.41**	0.21
2	E	10.11	4.96	0.26	0.67
3	E	10.31	5.96	0.17	0.54
7	E	10.64	5.31	0.16	0.62
9	E	13.99	1.62	0.05	0.95
**12**	N	1.49	3.96	**0.96**	0.41
**13**	N	2.82	1.23	**0.83**	0.54
**15**	N	5.63	5.25	**0.78**	0.16
**17**	N	3.43	6.06	**0.75**	0.42
**18**	N	3.1	4.03	**0.69**	0.13
**19**	N	4.96	6.06	**0.67**	0.2
11	N	6.3	0.01	0.51	0.99
14	N	3.43	2.96	0.49	0.57
16	N	6.83	2.96	0.45	0.4
20	N	5.92	3.37	0.31	0.5
**22**	H	4.96	4.96	**0.67**	0.67
**23**	H	5.16	8.97	**0.64**	0.26
**26**	H	6.3	5.3	**0.61**	0.51
**27**	H	6.5	4.96	**0.48**	0.55
**29**	H	6.63	8.97	**0.47**	0.35
**30**	H	8.3	6.16	**0.4**	0.41
21	H	7.44	6.83	0.28	0.45
24	H	9.98	3.62	0.27	0.61
25	H	9.31	6.16	0.23	0.41
28	H	7.11	9.17	0.21	0.33

Note: E = erotic, N = neutral, H = happy.
